# Author Correction: Characterising the loss-of-function impact of 5’ untranslated region variants in 15,708 individuals

**DOI:** 10.1038/s41467-021-21052-3

**Published:** 2021-02-02

**Authors:** Nicola Whiffin, Konrad J. Karczewski, Xiaolei Zhang, Sonia Chothani, Miriam J. Smith, D. Gareth Evans, Angharad M. Roberts, Nicholas M. Quaife, Sebastian Schafer, Owen Rackham, Jessica Alföldi, Anne H. O’Donnell-Luria, Laurent C. Francioli, Genome Aggregation Database Production Team, Irina M. Armean, Eric Banks, Louis Bergelson, Kristian Cibulskis, Ryan L. Collins, Kristen M. Connolly, Miguel Covarrubias, Beryl Cummings, Mark J. Daly, Stacey Donnelly, Yossi Farjoun, Steven Ferriera, Stacey Gabriel, Laura D. Gauthier, Jeff Gentry, Namrata Gupta, Thibault Jeandet, Diane Kaplan, Kristen M. Laricchia, Christopher Llanwarne, Eric V. Minikel, Ruchi Munshi, Benjamin M. Neale, Sam Novod, Nikelle Petrillo, Timothy Poterba, David Roazen, Valentin Ruano-Rubio, Andrea Saltzman, Kaitlin E. Samocha, Molly Schleicher, Cotton Seed, Matthew Solomonson, Jose Soto, Grace Tiao, Kathleen Tibbetts, Charlotte Tolonen, Christopher Vittal, Gordon Wade, Arcturus Wang, Qingbo Wang, Nicholas A. Watts, Ben Weisburd, Genome Aggregation Database Consortium, Carlos A. Aguilar Salinas, Tariq Ahmad, Christine M. Albert, Diego Ardissino, Gil Atzmon, John Barnard, Laurent Beaugerie, Emelia J. Benjamin, Michael Boehnke, Lori L. Bonnycastle, Erwin P. Bottinger, Donald W. Bowden, Matthew J. Bown, John C. Chambers, Juliana C. Chan, Daniel Chasman, Judy Cho, Mina K. Chung, Bruce Cohen, Adolfo Correa, Dana Dabelea, Mark J. Daly, Dawood Darbar, Ravindranath Duggirala, Josée Dupuis, Patrick T. Ellinor, Roberto Elosua, Jeanette Erdmann, Tõnu Esko, Martti Färkkilä, Jose Florez, Andre Franke, Gad Getz, Benjamin Glaser, Stephen J. Glatt, David Goldstein, Clicerio Gonzalez, Leif Groop, Christopher Haiman, Craig Hanis, Matthew Harms, Mikko Hiltunen, Matti M. Holi, Christina M. Hultman, Mikko Kallela, Jaakko Kaprio, Sekar Kathiresan, Bong-Jo Kim, Young Jin Kim, George Kirov, Jaspal Kooner, Seppo Koskinen, Harlan M. Krumholz, Subra Kugathasan, Soo Heon Kwak, Markku Laakso, Terho Lehtimäki, Ruth J. F. Loos, Steven A. Lubitz, Ronald C. W. Ma, Jaume Marrugat, Kari M. Mattila, Steven McCarroll, Mark I. McCarthy, Dermot McGovern, Ruth McPherson, James B. Meigs, Olle Melander, Andres Metspalu, Benjamin M. Neale, Peter M. Nilsson, Michael C. O’Donovan, Dost Ongur, Lorena Orozco, Michael J. Owen, Colin N. A. Palmer, Aarno Palotie, Kyong Soo Park, Carlos Pato, Ann E. Pulver, Nazneen Rahman, Anne M. Remes, John D. Rioux, Samuli Ripatti, Dan M. Roden, Danish Saleheen, Veikko Salomaa, Nilesh J. Samani, Jeremiah Scharf, Heribert Schunkert, Moore B. Shoemaker, Pamela Sklar, Hilkka Soininen, Harry Sokol, Tim Spector, Patrick F. Sullivan, Jaana Suvisaari, E. Shyong Tai, Yik Ying Teo, Tuomi Tiinamaija, Ming Tsuang, Dan Turner, Teresa Tusie-Luna, Erkki Vartiainen, Hugh Watkins, Rinse K. Weersma, Maija Wessman, James G. Wilson, Ramnik J. Xavier, Marquis P. Vawter, Stuart A. Cook, Paul J. R. Barton, Daniel G. MacArthur, James S. Ware

**Affiliations:** 1 National Heart and Lung Institute and MRC London Institute of Medical Sciences, Imperial College London, Du Cane Road, London, W12 0NN UK; 2 NIHR Royal Brompton Cardiovascular Research Centre, Royal Brompton and Harefield National Health Service Foundation Trust, Sydney Street, London, SW3 6NP UK; 3 Medical and Population Genetics, Broad Institute of MIT and Harvard, 415 Main Street, Cambridge, MA 02142 USA; 4 Analytical and Translational Genetics Unit, Massachusetts General Hospital, 55 Fruit Street, Boston, MA 02114 USA; 5 Program in Cardiovascular and Metabolic Disorders, Duke-NUS Medical School, 8 College Road, Singapore, 169857 Singapore; 6 NW Genomic Laboratory Hub, Centre for Genomic Medicine, Division of Evolution and Genomic Science, St Mary’s Hospital, University of Manchester, Oxford Road, Manchester, M13 9WL UK; 7 National Heart Centre Singapore, 5 Hospital Drive, Singapore, 169609 Singapore; 8 Division of Genetics and Genomics, Boston Children’s Hospital, Boston, MA 02115 USA; 9 Department of Pediatrics, Harvard Medical School, Boston, MA 02115 USA; 10 Centre for Population Genomics, Garvan Institute of Medical Research, and UNSW Sydney, Sydney, Australia; 11 Centre for Population Genomics, Murdoch Children’s Research Institute, Melbourne, Australia; 12 European Molecular Biology Laboratory, European Bioinformatics Institute, Wellcome Genome Campus, Hinxton, Cambridge CB10 1SD UK; 13 Data Sciences Platform, Broad Institute of MIT and Harvard, Cambridge, MA 02142 USA; 14 Center for Genomic Medicine, Massachusetts General Hospital, Boston, MA 02114 USA; 15 Program in Bioinformatics and Integrative Genomics, Harvard Medical School, Boston, MA 02115 USA; 16 Genomics Platform, Broad Institute of MIT and Harvard, Cambridge, MA 02142 USA; 17 Program in Biological and Biomedical Sciences, Harvard Medical School, Boston, MA 02115 USA; 18 Stanley Center for Psychiatric Research, Broad Institute of MIT and Harvard, Cambridge, MA 02142 USA; 19 Broad Genomics, Broad Institute of MIT and Harvard, Cambridge, MA 02142 USA; 20 Wellcome Sanger Institute, Wellcome Genome Campus, Hinxton, Cambridge, CB10 1SA UK; 21 Unidad de Investigacion de Enfermedades Metabolicas, Instituto Nacional de Ciencias Medicas y Nutricion, Mexico City, Mexico; 22 Peninsula College of Medicine and Dentistry, Exeter, EX2 4TH UK; 23 Division of Preventive Medicine, Brigham and Women’s Hospital, Boston, MA 02115 USA; 24 Division of Cardiovascular Medicine, Brigham and Women’s Hospital and Harvard Medical School, Boston, MA 02115 USA; 25 Department of Cardiology University Hospital, 43100 Parma, Italy; 26 Department of Biology, Faculty of Natural Sciences, University of Haifa, 3498838 Haifa, Israel; 27 Departments of Medicine and Genetics, Albert Einstein College of Medicine, Bronx, NY 10461 USA; 28 Department of Quantitative Health Sciences, Lerner Research Institute, Cleveland Clinic, Cleveland, OH 44122 USA; 29 APHP, Gastroenterology Department, Saint Antoine Hospital, Sorbonne Université, Paris, France; 30 NHLBI and Boston University’s Framingham Heart Study, Framingham, MA 01701 USA; 31 Department of Medicine, Boston University School of Medicine, Boston, MA 02118 USA; 32 Department of Epidemiology, Boston University School of Public Health, Boston, MA 02118 USA; 33 Department of Biostatistics and Center for Statistical Genetics, University of Michigan, Ann Arbor, MI 48109 USA; 34 National Human Genome Research Institute, National Institutes of Health, Bethesda, MD 20892 USA; 35 The Charles Bronfman Institute for Personalized Medicine, Icahn School of Medicine at Mount Sinai, New York, NY 10029 USA; 36 Department of Biochemistry, Wake Forest School of Medicine, Winston-Salem, NC 27101 USA; 37 Center for Genomics and Personalized Medicine Research, Wake Forest School of Medicine, Winston-Salem, NC 27101 USA; 38 Center for Diabetes Research, Wake Forest School of Medicine, Winston-Salem, NC 27101 USA; 39 Department of Cardiovascular Sciences, University of Leicester, Leicester, LE1 7RH UK; 40 NIHR Leicester Biomedical Research Centre, Glenfield Hospital, Leicester, LE3 9QP UK; 41 Department of Epidemiology and Biostatistics, Imperial College London, London, SW7 2BX UK; 42 Department of Cardiology, Ealing Hospital NHS Trust, Southall, UB1 3HW UK; 43 Imperial College Healthcare NHS Trust, Imperial College London, London, SW7 2BX UK; 44 Department of Medicine and Therapeutics, The Chinese University of Hong Kong, 999077 Hong Kong, China; 45 Department of Medicine, Harvard Medical School, Boston, MA 02115 USA; 46 Departments of Cardiovascular Medicine, Cellular and Molecular Medicine, Molecular Cardiology, and Quantitative Health Sciences, Cleveland Clinic, Cleveland, OH 44195 USA; 47 McLean Hospital, Belmont, MA 02478 USA; 48 Department of Medicine, University of Mississippi Medical Center, Jackson, MS 39216 USA; 49 Department of Epidemiology, Colorado School of Public Health, Aurora, Colorado 80246 USA; 50 Department of Medicine and Pharmacology, University of Illinois at Chicago, Chicago, IL 60607 USA; 51 Department of Genetics, Texas Biomedical Research Institute, San Antonio, TX 78227 USA; 52 Department of Biostatistics, Boston University School of Public Health, Boston, MA 02118 USA; 53 National Heart, Lung, and Blood Institute’s Framingham Heart Study, Framingham, MA 01702 USA; 54 Cardiac Arrhythmia Service and Cardiovascular Research Center, Massachusetts General Hospital, Boston, MA 02114 USA; 55 Cardiovascular Epidemiology and Genetics, Hospital del Mar Medical Research Institute (IMIM), Barcelona, Catalonia Spain; 56 CIBER CV, Barcelona, Catalonia Spain; 57 Departament of Medicine, Medical School, University of Vic-Central University of Catalonia, Catalonia, 08500 Spain; 58 Institute for Cardiogenetics, University of Lübeck, Lübeck, 23562 Germany; 59 DZHK (German Research Centre for Cardiovascular Research) partner site Hamburg/Lübeck/Kiel, 23562 Lübeck, Germany; 60 University Heart Center Lübeck, 23562 Lübeck, Germany; 61 Estonian Genome Center, Institute of Genomics, University of Tartu, Tartu, 50090 Estonia; 62 Clinic of Gastroenterology, Helsinki University and Helsinki University Hospital, FL 00014 Helsinki, Finland; 63 Diabetes Unit and Center for Genomic Medicine, Massachusetts General Hospital; Programs in Metabolism and Medical and Population Genetics, Broad Institute; Department of Medicine, Harvard Medical School, Boston, MA 02115 USA; 64 Institute of Clinical Molecular Biology (IKMB), Christian-Albrechts-University of Kiel, Kiel, 24118 Germany; 65 Bioinformatics Program, Department of Pathology, MGH Cancer Center, Boston, 02114 USA; 66 Cancer Genome Computational Analysis, Broad Institute, Cambridge, 02142 USA; 67 Endocrinology and Metabolism Department, Hadassah-Hebrew University Medical Center, Jerusalem, Israel; 68 Department of Psychiatry and Behavioral Sciences, SUNY Upstate Medical University, Syracuse, NY 13421 USA; 69 Institute for Genomic Medicine, Columbia University Medical Center, Hammer Health Sciences, 1408, 701 West 168th Street, New York, NY 10032 USA; 70 Department of Genetics & Development, Columbia University Medical Center, Hammer Health Sciences, 1602, 701 West 168th Street, New York, NY 10032 USA; 71 Centro de Investigacion en Salud Poblacional Instituto Nacional de Salud Publica MEXICO, Cuernavace, 62100 Mexico; 72 Lund University, SE-221 00 Lund, Sweden; 73 Institute for Molecular Medicine Finland (FIMM), HiLIFE, University of Helsinki, FL 00014 Helsinki, Finland; 74 Lund University Diabetes Centre, SE-221 00 Lund, Sweden; 75 Human Genetics Center, University of Texas Health Science Center at Houston, Houston, TX 77030 USA; 76 Department of Neurology, Columbia University, New York, 10032 USA; 77 Institute of Genomic Medicine, Columbia University, New York, 10032 USA; 78 Institute of Biomedicine, University of Eastern Finland, Kuopio, FI-80101 Finland; 79 Department of Psychiatry, PL 320, Helsinki University Central Hospital, Lapinlahdentie, 00 180, Helsinki, Finland; 80 Department of Medical Epidemiology and Biostatistics, Karolinska Institutet, Stockholm, 171 77 Sweden; 81 Icahn School of Medicine at Mount Sinai, New York, NY 10029 USA; 82 Department of Neurology, Helsinki University Central Hospital, Fl 00290 Helsinki, Finland; 83 Department of Public Health, Faculty of Medicine, University of Helsinki, FL 00014 Helsinki, Finland; 84 Center for Genomic Medicine, Massachusetts General Hospital, Boston, MA 02114 USA; 85 Cardiovascular Disease Initiative and Program in Medical and Population Genetics, Broad Institute of MIT and Harvard, Cambridge, MA 02142 USA; 86 Center for Genome Science, Korea National Institute of Health, Chungcheongbuk-do, Republic of Korea; 87 MRC Centre for Neuropsychiatric Genetics and Genomics, Cardiff University School of Medicine, Hadyn Ellis Building, Maindy Road, Cardiff, CF24 4HQ UK; 88 National Heart and Lung Institute, Cardiovascular Sciences, Imperial College London, Hammersmith Campus, London, SW7 2BX UK; 89 Department of Health, THL-National Institute for Health and Welfare, 00271 Helsinki, Finland; 90 Section of Cardiovascular Medicine, Department of Internal Medicine, Yale School of Medicine, New Haven, CT 06510 USA; 91 Division of Pediatric Gastroenterology, Emory University School of Medicine, Atlanta, GA 30322 USA; 92 Department of Internal Medicine, Seoul National University Hospital, Seoul, Republic of Korea; 93 Institute of Clinical Medicine, The University of Eastern Finland, Kuopio, Finland; 94 Kuopio University Hospital, Kuopio, 70210 Finland; 95 Department of Clinical Chemistry, Fimlab Laboratories and Finnish Cardiovascular Research Center-Tampere, Faculty of Medicine and Health Technology, Tampere University, FI-33014 Tampere, Finland; 96 The Mindich Child Health and Development Institute, Icahn School of Medicine at Mount Sinai, New York, NY 10029 USA; 97 Li Ka Shing Institute of Health Sciences, The Chinese University of Hong Kong, Hong Kong, China; 98 Hong Kong Institute of Diabetes and Obesity, The Chinese University of Hong Kong, Hong Kong, China; 99 Cardiovascular Research REGICOR Group, Hospital del Mar Medical Research Institute (IMIM), Barcelona, 08003 Catalonia Spain; 100 Department of Genetics, Harvard Medical School, Boston, MA 02115 USA; 101 Oxford Centre for Diabetes, Endocrinology and Metabolism, University of Oxford, Churchill Hospital, Old Road, Headington, Oxford, OX3 7LJ UK; 102 Wellcome Centre for Human Genetics, University of Oxford, Roosevelt Drive, Oxford, OX3 7BN UK; 103 Oxford NIHR Biomedical Research Centre, Oxford University Hospitals NHS Foundation Trust, John Radcliffe Hospital, Oxford, OX3 9DU UK; 104 F Widjaja Foundation Inflammatory Bowel and Immunobiology Research Institute, Cedars-Sinai Medical Center, Los Angeles, CA 90048 USA; 105 Atherogenomics Laboratory, University of Ottawa Heart Institute, Ottawa, ON K1Y 4W7 Canada; 106 Division of General Internal Medicine, Massachusetts General Hospital, Boston, MA 02114 USA; 107 Program in Population and Medical Genetics, Broad Institute, Cambridge, MA 02142 USA; 108 Department of Clinical Sciences, University Hospital Malmo Clinical Research Center, Lund University, SE-221 00 Malmo, Sweden; 109 Department of Clinical Sciences, Lund University, Skane University Hospital, SE-221 00 Malmo, Sweden; 110 Instituto Nacional de Medicina Genómica (INMEGEN), Mexico City, 14610 Mexico; 111 Medical Research Institute, Ninewells Hospital and Medical School, University of Dundee, Dundee, DD1 4HN UK; 112 Department of Molecular Medicine and Biopharmaceutical Sciences, Graduate School of Convergence Science and Technology, Seoul National University, Seoul, Republic of Korea; 113 Department of Psychiatry, Keck School of Medicine at the University of Southern California, Los Angeles, CA 90033 USA; 114 Department of Psychiatry and Behavioral Sciences, Johns Hopkins University School of Medicine, Baltimore, MD 20105 USA; 115 Division of Genetics and Epidemiology, Institute of Cancer Research, London, SM2 5NG UK; 116 Medical Research Center, Oulu University Hospital, Oulu, Finland and Research Unit of Clinical Neuroscience, Neurology, University of Oulu, FI-90014 Oulu, Finland; 117 Research Center, Montreal Heart Institute, Montreal, QC H1T 1C8 Canada; 118 Department of Medicine, Faculty of Medicine, Université de Montréal, Montréal, H3T 1J4 Québec Canada; 119 Broad Institute of MIT and Harvard, Cambridge, MA 02142 USA; 120 Department of Biomedical Informatics, Vanderbilt University Medical Center, Nashville, TN 37212 USA; 121 Department of Medicine, Vanderbilt University Medical Center, Nashville, TN 37212 USA; 122 Department of Biostatistics and Epidemiology, Perelman School of Medicine at the University of Pennsylvania, Philadelphia, PA 19104 USA; 123 Department of Medicine, Perelman School of Medicine at the University of Pennsylvania, Philadelphia, PA 19104 USA; 124 Center for Non-Communicable Diseases, Karachi, 75300 Pakistan; 125 National Institute for Health and Welfare, Helsinki, FI 00271 Finland; 126 Deutsches Herzzentrum München, München, 80636 Germany; 127 Technische Universität München, München, 80333 Germany; 128 Division of Cardiovascular Medicine, Nashville VA Medical Center and Vanderbilt University, School of Medicine, Nashville, TN 37232-8802 USA; 129 Department of Psychiatry, Icahn School of Medicine at Mount Sinai, New York, NY 10029 USA; 130 Department of Genetics and Genomic Sciences, Icahn School of Medicine at Mount Sinai, New York, NY 10029 USA; 131 Institute for Genomics and Multiscale Biology, Icahn School of Medicine at Mount Sinai, New York, NY 10029 USA; 132 Institute of Clinical Medicine, neurology, University of Eastern Finland, Kuopio, Finland; 133 Department of Twin Research and Genetic Epidemiology, King’s College London, London, SE1 7EH UK; 134 Departments of Genetics and Psychiatry, University of North Carolina, Chapel Hill, NC 27599 USA; 135 Saw Swee Hock School of Public Health, National University of Singapore, National University Health System, Singapore, 117549 Singapore; 136 Department of Medicine, Yong Loo Lin School of Medicine, National University of Singapore, Singapore, 117597 Singapore; 137 Duke-NUS Graduate Medical School, Singapore, 169857 Singapore; 138 Life Sciences Institute, National University of Singapore, Singapore, 117456 Singapore; 139 Department of Statistics and Applied Probability, National University of Singapore, Singapore, 119077 Singapore; 140 Folkhälsan Institute of Genetics, Folkhälsan Research Center, Helsinki, Finland; 141 HUCH Abdominal Center, Helsinki University Hospital, Helsinki, 00260 Finland; 142 Center for Behavioral Genomics, Department of Psychiatry, University of California, San Diego, CA 94720 USA; 143 Institute of Genomic Medicine, University of California, San Diego, CA 94720 USA; 144 Juliet Keidan Institute of Pediatric Gastroenterology, Shaare Zedek Medical Center, The Hebrew University of Jerusalem, Jerusalem, Israel; 145 Instituto de Investigaciones Biomédicas UNAM, Mexico City, 04510 Mexico; 146 Instituto Nacional de Ciencias Médicas y Nutrición Salvador Zubirán Mexico City, Mexico City, 14080 Mexico; 147 Radcliffe Department of Medicine, University of Oxford, Oxford, OX3 9DU UK; 148 Department of Gastroenterology and Hepatology, University of Groningen and University Medical Center Groningen, Groningen, 9713 GZ The Netherlands; 149 Department of Physiology and Biophysics, University of Mississippi Medical Center, Jackson, MS 39216 USA; 150 Program in Infectious Disease and Microbiome, Broad Institute of MIT and Harvard, Cambridge, MA 02142 USA; 151 Center for Computational and Integrative Biology, Massachusetts General Hospital, Boston, MA 02114 USA; 152 Department of Psychiatry & Human Behavior, University of California Irvine, Irvine, CA USA

**Keywords:** Genome informatics, Genomics, Medical genomics, Genetics research

Correction to: *Nature Communications* 10.1038/s41467-019-10717-9, published online 27 May 2020.

The original version of this Article omitted from the Genome Aggregation Database consortium the member Marquis P. Vawter, from the Department of Psychiatry & Human Behavior, University of California Irvine, Irvine, CA, USA. Additionally, the following was added to the Author Contributions: ‘All authors listed under The Genome Aggregation Database Consortium contributed to the generation of the primary data incorporated into the gnomAD resource’. This has been corrected in both the PDF and HTML versions of the Article.

