# Supplementary material for: Characterising the loss-of-function impact of 5’ untranslated region variants in 15,708 individuals
Source: Nat Commun. 2020 May 27;11:2523. doi: 10.1038/s41467-019-10717-9 (PMC7253449; doi:10.1038/s41467-019-10717-9)
Supplement: Supplementary file 1 — Supplementary Information [file 41467_2019_10717_MOESM1_ESM.docx]

**Supplementary Table 1**: uAUG-creating variants catalogued as disease mutations (DM) in HGMD[^1^](https://paperpile.com/c/FfThFv/fGPG) or (Likely) Pathogenic in ClinVar[^2^](https://paperpile.com/c/FfThFv/GzVS). All positions are in GRCh37.

| variant | Kozak strength | effect | gene | gene class | distance to start | clinvar | PubMed IDs |
| --- | --- | --- | --- | --- | --- | --- | --- |
| 1-43424429-C-T | Strong | out-of-frame oORF | SLC2A1 | 8 (High) | 107 |  | 28378819 |
| 1-209975332-G-T | Moderate | out-of-frame oORF | IRF6 | 8  (High) | 19 |  | 19282774 |
| 1-209975361-T-A | Moderate | out-of-frame oORF | IRF6 | 8  (High) | 49 |  | 12219090 |
| 1-209979367-C-T | Strong | out-of-frame oORF | IRF6 | 8  (High) | 151 |  | 19282774 |
| 5-14871567-G-A | Moderate | CDS elongated | ANKH | 8  (High) | 12 |  | 12297987 |
| 5-36877266-C-T | Weak | uORF created | NIPBL | 8  (High) | 95 |  | 17661813 |
| 6-137143759-C-T | Strong | out-of-frame oORF | PEX7 | 8  (High) | 46 | Phytanic_acid_storage_disease;Pathogenic;38871 | 12325024 |
| 7-107301244-A-G | Moderate | uORF created | SLC26A4 | 8  (High) | 62 |  | 19204907 |
| 7-117120115-C-T | Moderate | out-of-frame oORF | CFTR | 8  (High) | 35 |  | 21837768 |
| 9-21974860-C-A | Strong | out-of-frame oORF | CDKN2A | 8  (High) | 35 | Hereditary_cancer-predisposing_syndrome\|Hereditary_cutaneous_melanoma\|Melanoma-pancreatic_cancer_syndrome;Pathogenic;182414 | 9916806 |
| 9-130616761-G-A | Moderate | out-of-frame oORF | ENG | 8  (High) | 128 | Osler_hemorrhagic_telangiectasia_syndrome;Pathogenic;407113 | 21967607 |
| 9-133327612-C-T | Moderate | out-of-frame oORF | ASS1 | 3 (Low) | 5 | Citrullinemia_type_I;Likely_pathogenic;203632 | 19006241 |
| 11-5248280-C-T | Moderate | out-of-frame oORF | HBB | 8 (High) | 29 | beta_Thalassemia;Pathogenic;393702 | 1717406 |
| 11-17409692-G-A | Moderate | out-of-frame oORF | KCNJ11 | 3 (Low) | 55 |  | 12364426 |
| 14-55369403-G-A | Moderate | out-of-frame oORF | GCH1 | 8 (High) | 23 |  | 10825351 |
| 17-29422056-G-A | Moderate | out-of-frame oORF | NF1 | 8 (High) | 272 |  | 27322474 |
| 17-70117348-G-A | Moderate | out-of-frame oORF | SOX9 | 8 (High) | 185 |  | 28546996 |
| 19-11200076-C-A | Weak | uORF created | LDLR | 8 (High) | 149 | Familial_hypercholesterolemia;Likely_pathogenic;250946 | 22698793 |
| 19-11200127-C-T | Moderate | uORF created | LDLR | 8 (High) | 99 | Familial_hypercholesterolemia;Pathogenic;440535 | NA |
| 19-11200128-G-A | Strong | out-of-frame oORF | LDLR | 8 (High) | 97 | Familial_hypercholesterolemia;Likely_pathogenic;430743 | NA |
| 19-11200202-AC-A | Moderate | uORF created | LDLR | 8 (High) | 22 |  | 25248394 |
| 22-50523373-G-A | Moderate | out-of-frame oORF | MLC1 | 8 (High) | 43 |  | 25497041 |
| X-38211811-A-G | Weak | uORF created | OTC | 8 (High) | 141 | Ornithine_carbamoyltransferase_deficiency;Likely_pathogenic;487341 | NA |
| X-49114969-C-A | Moderate | out-of-frame oORF | FOXP3 | 8 (High) | 8 |  | 16371377 |
| X-148579835-ATG-A | Moderate | uORF created | IDS | 3 (Low) | 124 | Mucopolysaccharidosis,_MPS-II;Pathogenic;10496 | 1303211 |
| X-154250832-T-C | Moderate | out-of-frame oORF | F8 | 8 (High) | 7 |  | 22958177 |
| 2-96931137-G-A | Strong | out-of-frame oORF | TMEM127 | 7 (High) | 19 | Pheochromocytoma;Likely_pathogenic;126961 | 21156949 |
| 2-157189174-G-A | Moderate | uORF created | NR4A2 | 7 (High) | 310 |  | 19429166 |
| 4-6271704-G-T | Moderate | out-of-frame oORF | WFS1 | 7 (High) | 44 |  | 27395765 |
| 5-147211193-G-A | Moderate | uORF created | SPINK1 | 7 (High) | 54 |  | 10835640, 27171515, 26228362, 21610753 |
| 7-143013247-C-A | Strong | out-of-frame oORF | CLCN1 | 7 (High) | 59 |  | 23771340 |
| 12-121416385-C-T | Moderate | uORF created | HNF1A | 7 (High) | 188 |  | 10649494 |
| 17-66508599-G-A | Strong | out-of-frame oORF | PRKAR1A | 7 (High) | 97 |  | 12424709 |
| 2-25387652-G-T | Moderate | out-of-frame oORF | POMC | 6 (Mod) | 11 | Proopiomelanocortin_deficiency;Pathogenic;13355 | 9620771, 27906547, 23649472 |
| 6-26087649-G-A | Moderate | out-of-frame oORF | HFE | 6 (Mod) | 20 |  | 21175851 |
| 22-19710933-C-G | Moderate | CDS elongated | GP1BB | 6 (Mod) | 162 | Bernard-Soulier_syndrome,_type_B;Pathogenic;16041 | 8703016 |
| 11-299504-G-A | Strong | CDS elongated | IFITM5 | 5 (Low) | 15 | Osteogenesis_imperfecta_type_5;Pathogenic;37143 | 22863190 |
| 19-35773456-G-A | Strong | out-of-frame oORF | HAMP | 3 (Low) | 25 |  | 15198949 |
| 1-151372055-G-A | Moderate | CDS elongated | PSMB4 | 4 (Low) | 9 | PROTEASOME-ASSOCIATED_AUTOINFLAMMATORY_SYNDROME_3;Pathogenic;548956 | 28848544 |

**Supplementary Table 2**: Stop-removing variants catalogued as disease mutations (DM) in HGMD[^1^](https://paperpile.com/c/FfThFv/fGPG) or (Likely) Pathogenic in ClinVar[^2^](https://paperpile.com/c/FfThFv/GzVS).

| variant | Kozak strength | effect | gene | gene class | evidence of translation | clinvar | PubMed IDs |
| --- | --- | --- | --- | --- | --- | --- | --- |
| 4-159593534-A-G | Strong | out-of-frame oORF | ETFDH | 8 (High) | N |  | 23628458 |
| 8-21988118-T-C | Moderate | CDS elongated | HR | 8 (High) | Y | Hypotrichosis_4;Pathogenic;7344 | NA |
| 17-29422055-A-C | Strong | out-of-frame oORF | NF1 | 8 (High) | Y |  | 27322474 |
| X-68049525-T-C | Strong | out-of-frame oORF | EFNB1 | 8 (High) | N |  | 23335590 |

**Supplementary Table 3**: Additional phenotypic information for individuals diagnosed with neurofibromatosis type 2. VS - Vestibular schwannoma

| Individual | Family | Sex | Age symptoms | Age meningioma | Number meningiomas | Age VS | VS side | Number spinal tumours |
| --- | --- | --- | --- | --- | --- | --- | --- | --- |
| 201 | B | Female | 5 | NA | 0 | 41 | bilateral | 2 |
| 301 | B | Female | 20 | NA | 0 | 29 | bilateral | 2 |
| 202 | B | Male | 26 | 26 | 4 | 31 | left | 0 |
| 301 | A | Female | 32 | NA | 0 | 34 | Bilateral | 5 |
| 201 | A | Male | 56 | 57 | 3 | 57 | bilateral | 0 |


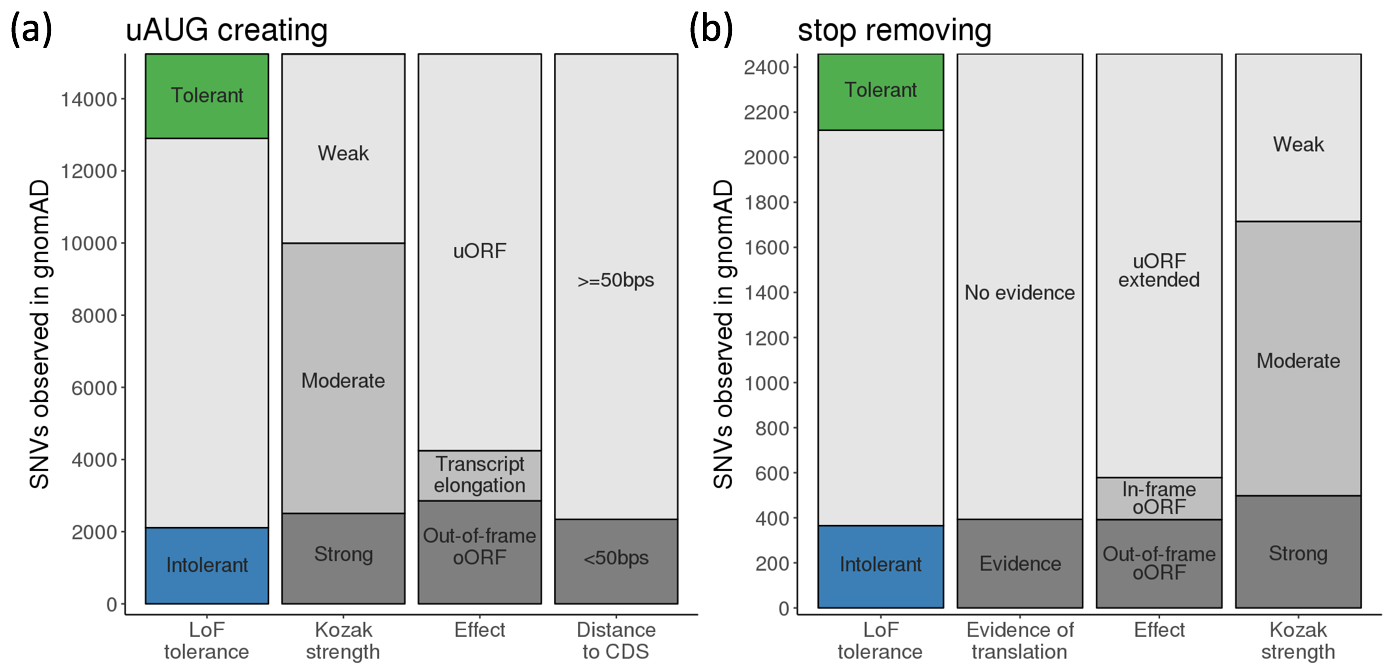


**Supplementary Figure 1**: Observed numbers of uAUG-creating and stop-removing SNVs in gnomAD[^3^](https://paperpile.com/c/FfThFv/yybq).


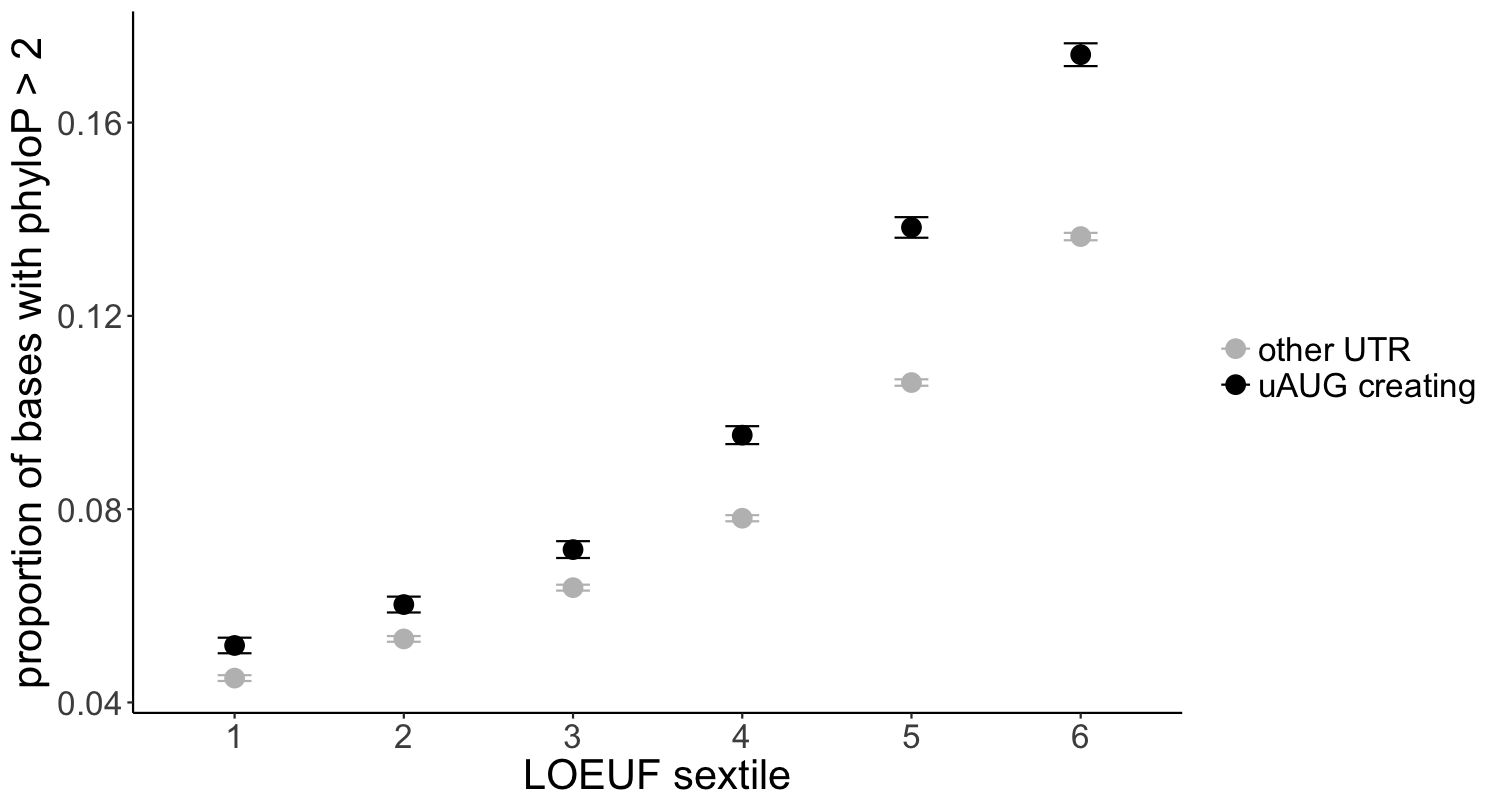


**Supplementary Figure 2**: The proportion of UTR bases at which a single base change could create an uAUG (black; n=534,296) and all other 5’UTR bases (grey; n=3,898,284) with phyloP[^4^](https://paperpile.com/c/FfThFv/2ENn) scores > 2. Bases are split into sextiles of LOEUF score[^3^](https://paperpile.com/c/FfThFv/yybq) of the corresponding genes, with 1 representing genes most tolerant to pLoF variants and 6 the most intolerant.


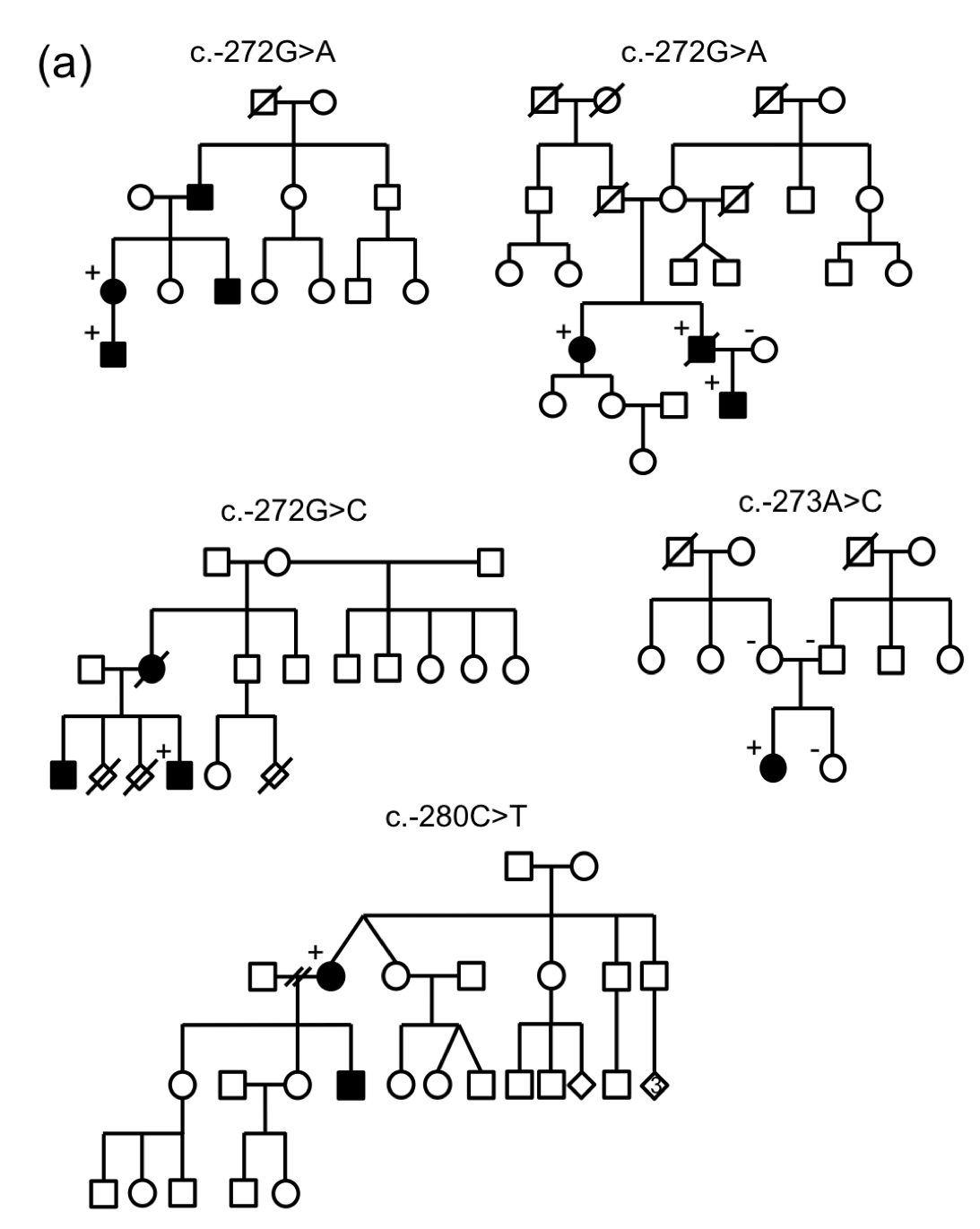


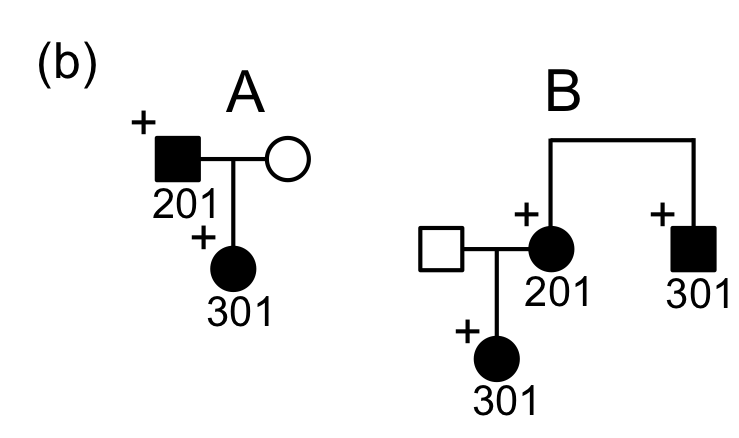


**Supplementary Figure 3**: Pedigrees for families with (a) *NF1*[*^5^*](https://paperpile.com/c/FfThFv/UCZm) and (b) *NF2* variants. Affected individuals are shown by shaded symbols. Genetically tested individuals are marked with either a + or a - representing variant carriers and non-carriers respectively. For *NF2* families, each individual is numbered to correspond with rows of phenotypic information in Supplementary Table 3.


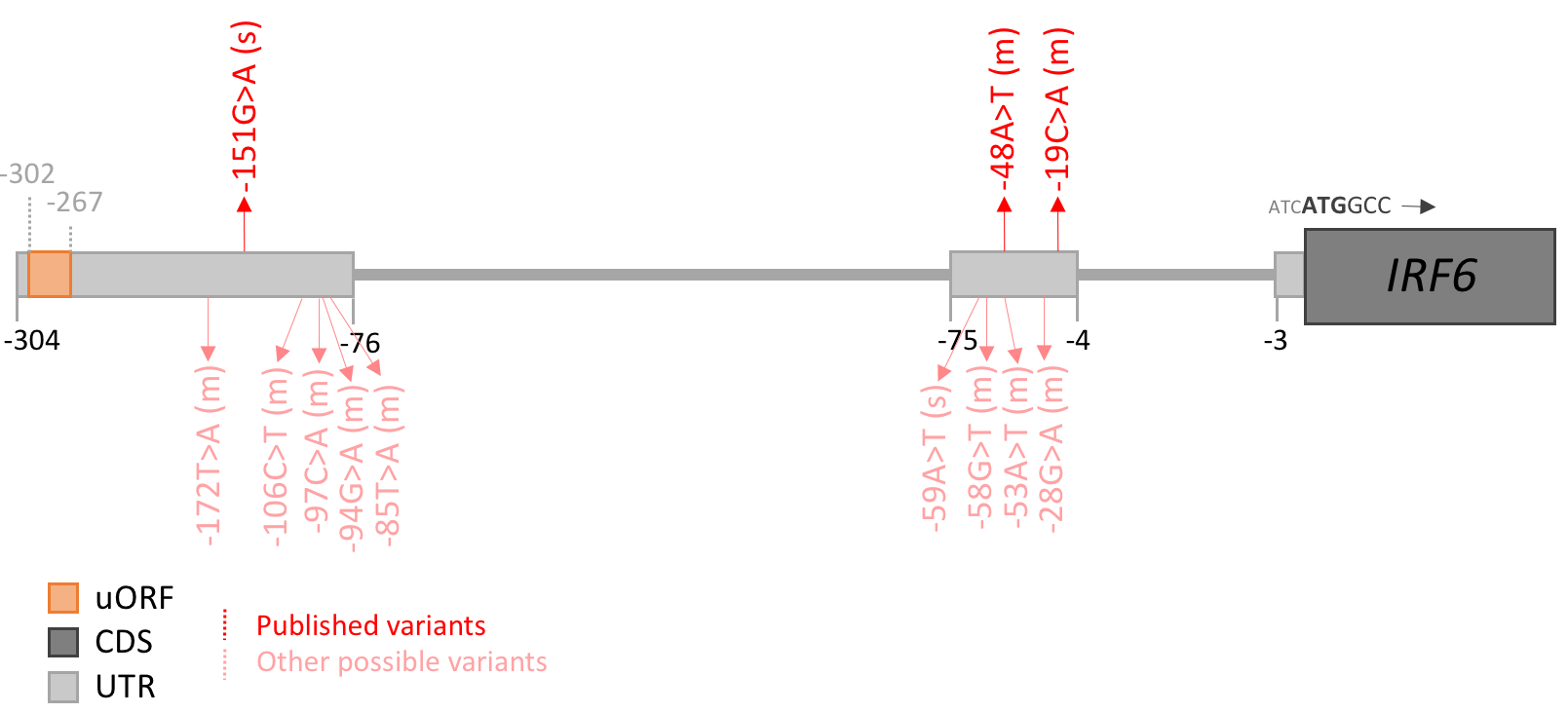


**Supplementary Figure 4**: Schematic of the 5’UTR of *IRF6* showing the location of uAUG-creating variants identified by de Lima *et al*.[^6^](https://paperpile.com/c/FfThFv/BobO) (bright red) and all other possible uAUG-creating SNVs (faded red) which would be created into a strong or moderate Kozak consensus and form an out-of-frame oORF. The strength of the Kozak consensus is shown in brackets (“s” for strong, “m” for moderate).


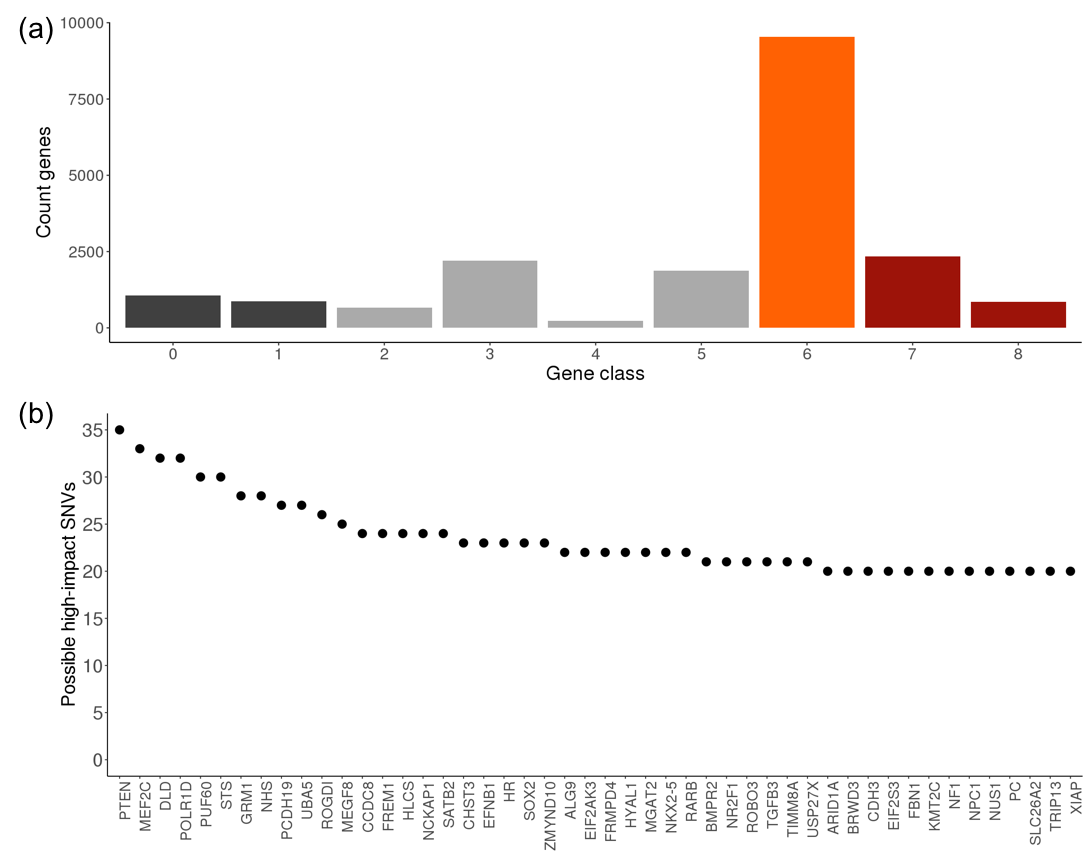


**Supplementary Figure 5**: Identifying genes where there is a high likelihood that uORF-perturbing variants will be deleterious. (a) Plot of all 18,593 by category (see methods) coloured by unknown (dark grey), low (light grey), moderate (orange) and high likelihood (dark red) that uORF-perturbing variants will be deleterious. (b) Genes in class 8 (not classified with a ‘Low’ likelihood and where LoF and/or haploinsufficiency is a known mechanism of human disease) with ≥20 possible high-impact uAUG-creating and stop-removing SNVs.

**Supplementary References**

1. [Krawczak, M., Ball, E. V., Stenson, P. & Cooper, D. N. HGMD: The Human Gene Mutation Database. *Bioinformatics: Databases and Systems* 99–104 doi:](http://paperpile.com/b/FfThFv/fGPG)[10.1007/0-306-46903-0_9](http://dx.doi.org/10.1007/0-306-46903-0_9)

2. [Landrum, M. J. *et al.* ClinVar: public archive of interpretations of clinically relevant variants. *Nucleic Acids Res.* **44**, D862–8 (2016).](http://paperpile.com/b/FfThFv/GzVS)

3. [Karczewski, K. J. *et al.* Variation across 141,456 human exomes and genomes reveals the spectrum of loss-of-function intolerance across human protein-coding genes: Supplementary Information. *Genomics* (2019).](http://paperpile.com/b/FfThFv/yybq)

4. [Pollard, K. S., Hubisz, M. J., Rosenbloom, K. R. & Siepel, A. Detection of nonneutral substitution rates on mammalian phylogenies. *Genome Res.* **20**, 110–121 (2010).](http://paperpile.com/b/FfThFv/2ENn)

5. [Evans, D. G. *et al.* Comprehensive RNA Analysis of the NF1 Gene in Classically Affected NF1 Affected Individuals Meeting NIH Criteria has High Sensitivity and Mutation Negative Testing is Reassuring in Isolated Cases With Pigmentary Features Only. *EBioMedicine* **7**, 212–220 (2016).](http://paperpile.com/b/FfThFv/UCZm)

6. [de Lima, R. L. L. F. *et al.* Prevalence and nonrandom distribution of exonic mutations in interferon regulatory factor 6 in 307 families with Van der Woude syndrome and 37 families with popliteal pterygium syndrome. *Genet. Med.* **11**, 241–247 (2009).](http://paperpile.com/b/FfThFv/BobO)

**Supplementary Data 1**: Genes with ≥10 possible predicted high-impact uAUG-creating or stop-removing SNVs, and for which LoF and/or haploinsufficiency is a known mechanism of human disease (either curated as haploinsufficient, curated as acting via a LoF mechanism in DDG2P or with ≥10 high-confidence pathogenic LoF variants documented in ClinVar).
