# Supplementary material for: Characterising the loss-of-function impact of 5’ untranslated region variants in 15,708 individuals
Source: Nat Commun. 2020 May 27;11:2523. doi: 10.1038/s41467-019-10717-9 (PMC7253449; doi:10.1038/s41467-019-10717-9)
Supplement: Supplementary file 3 — Reporting Summary [file 41467_2019_10717_MOESM3_ESM.pdf]

nature research

Corresponding author(s): Nicola Whiffin

Last updated by author(s): May 13, 2019

Reporting Summary

Nature Research wishes to improve the reproducibility of the work that we publish. This form provides structure for consistency and transparency in reporting. For further information on Nature Research policies, see Authors & Referees and the Editorial Policy Checklist.

Statistics

For all statistical analyses, confirm that the following items are present in the figure legend, table legend, main text, or Methods section.

n/a	Confirmed	
☐	☒	The exact sample size (*n*) for each experimental group/condition, given as a discrete number and unit of measurement
☐	☒	A statement on whether measurements were taken from distinct samples or whether the same sample was measured repeatedly
☐	☒	The statistical test(s) used AND whether they are one- or two-sided<br>*Only common tests should be described solely by name; describe more complex techniques in the Methods section.*
☐	☒	A description of all covariates tested
☐	☒	A description of any assumptions or corrections, such as tests of normality and adjustment for multiple comparisons
☐	☒	A full description of the statistical parameters including central tendency (e.g. means) or other basic estimates (e.g. regression coefficient) AND variation (e.g. standard deviation) or associated estimates of uncertainty (e.g. confidence intervals)
☐	☒	For null hypothesis testing, the test statistic (e.g. *F*, *t*, *r*) with confidence intervals, effect sizes, degrees of freedom and *P* value noted<br>*Give P values as exact values whenever suitable.*
☒	☐	For Bayesian analysis, information on the choice of priors and Markov chain Monte Carlo settings
☒	☐	For hierarchical and complex designs, identification of the appropriate level for tests and full reporting of outcomes
☐	☒	Estimates of effect sizes (e.g. Cohen's *d*, Pearson's *r*), indicating how they were calculated

Our web collection on statistics for biologists contains articles on many of the points above.

Software and code

Policy information about availability of computer code

Data collection	We have made available a custom plugin to the Ensembl variant effect predictor that annotates variants as in the manuscript at https://github.com/ImperialCardioGenetics/uORFs.
Data analysis	Data analysis and figure creation was done using custom Perl and R scripts. These will be made available in GitHub at https://github.com/ImperialCardioGenetics/uORFs.

For manuscripts utilizing custom algorithms or software that are central to the research but not yet described in published literature, software must be made available to editors/reviewers. We strongly encourage code deposition in a community repository (e.g. GitHub). See the Nature Research guidelines for submitting code & software for further information.

Data

Policy information about availability of data

All manuscripts must include a data availability statement. This statement should provide the following information, where applicable:

- Accession codes, unique identifiers, or web links for publicly available datasets
- A list of figures that have associated raw data
- A description of any restrictions on data availability

The following statement is included in the manuscript: "All possible uAUG-creating and stop-removing SNVs for canonical Gencode transcripts along with likelihood classifications for all genes are available for download at https://github.com/ImperialCardioGenetics/uORFs."

Field-specific reporting

Please select the one below that is the best fit for your research. If you are not sure, read the appropriate sections before making your selection.

☒ Life sciences ☐ Behavioural & social sciences ☐ Ecological, evolutionary & environmental sciences

For a reference copy of the document with all sections, see nature.com/documents/nr-reporting-summary-flat.pdf

Life sciences study design

All studies must disclose on these points even when the disclosure is negative.

Sample size	This study was opportunistic, and involved secondary use of all available data. No sample size was predetermined.
Data exclusions	All data exclusions are clearly described in the methods section.
Replication	We did not attempt to reproduce any findings in a separate dataset, instead all available data was used for discovery analyses.
Randomization	As this was a population-based study, no randomization was performed.
Blinding	Blinding was not relevant for this population-based study.

Reporting for specific materials, systems and methods

We require information from authors about some types of materials, experimental systems and methods used in many studies. Here, indicate whether each material, system or method listed is relevant to your study. If you are not sure if a list item applies to your research, read the appropriate section before selecting a response.

Materials & experimental systems

n/a	Involved in the study	
☒	☐	Antibodies
☒	☐	Eukaryotic cell lines
☒	☐	Palaeontology
☒	☐	Animals and other organisms
☐	☒	Human research participants
☒	☐	Clinical data

Methods

n/a	Involved in the study	
☒	☐	ChIP-seq
☒	☐	Flow cytometry
☒	☐	MRI-based neuroimaging

Human research participants

Policy information about studies involving human research participants

Population characteristics	As an opportunistic collection of data, the participants in this study were not selected based on age, gender, or genotypic information.
Recruitment	Data were aggregated as part of the genome aggregation database (gnomAD). This represents secondary analysis of available sequencing data and is subject to expected biases such as under representation of certain ethnic and socioeconomic groups relative to the general population.
Ethics oversight	The following ethics statement is not currently included but will be added during revision: "We have complied with all relevant ethical regulations. This study was overseen by the Broad Institute's Office of Research Subject Protection and the Partners Human Research Committee, and was given a determination of Not Human Subjects Research. Informed consent was obtained from all participants."

Note that full information on the approval of the study protocol must also be provided in the manuscript.
