# Supplementary material for: Characterising the loss-of-function impact of 5’ untranslated region variants in 15,708 individuals
Source: Nat Commun. 2020 May 27;11:2523. doi: 10.1038/s41467-019-10717-9 (PMC7253449; doi:10.1038/s41467-019-10717-9)
Supplement: Supplementary file 4 — Description of Additional Supplementary Files [file 41467_2019_10717_MOESM4_ESM.docx]

**Title: Supplementary Data 1**
**Description:** Genes with ≥10 possible predicted high-impact uAUG-creating or stop-removing SNVs, and for which LoF and/or haploinsufficiency is a known mechanism of human disease (either curated as haploinsufficient, curated as acting via a LoF mechanism in DDG2P or with ≥10 high-confidence pathogenic LoF variants documented in ClinVar).
